# Investigation of Recurrence Rate and Associated Risk Factors of Laryngeal Squamous Cell Carcinoma in Patients Undergoing Transoral Laser Microsurgery: A Retrospective Cohort Study

**DOI:** 10.22038/ijorl.2025.90331.4018

**Published:** 2026

**Authors:** Mohammad Amin Zaeim Yekeh, Aslan Ahmadi, Pegah Alizade Pahlavan, Mohammad Mahdi Salem

**Affiliations:** 1 *ENT and Head and Neck Research Center and Department, The Five Senses Health Institute, School of Medicine, Iran University of Medical Sciences, Tehran, Iran.*

**Keywords:** Laryngeal Neoplasms, Carcinoma, Squamous Cell, Laser Therapy, Recurrence, Smoking, Prognosis, Risk Factors

## Abstract

**Introduction:**

Laryngeal squamous cell carcinoma (SCC) remains a significant health challenge, with transoral laser microsurgery (TLM) emerging as a preferred treatment for early-stage disease. This retrospective cohort study investigates recurrence patterns, survival outcomes, and prognostic factors in patients treated with TLM, with a focus on the role of smoking exposure.

**Materials and Methods:**

A retrospective cohort study was conducted on 142 patients with laryngeal SCC treated with TLM at Rasoul Akram Hospital (2016–2022). Patient demographics, tumor characteristics, smoking history (Pack-Years), and follow-up data were analyzed. Recurrence rates and survival were assessed using Kaplan-Meier analysis, and risk factors were evaluated using univariate logistic regression and Cox proportional hazards models.

**Results:**

The recurrence rate was 26.1% (n=37), predominantly local (70.3%), with a median time to recurrence of 15 months. Smoking exposure significantly correlated with recurrence risk (OR=1.02, 95% CI: 1.00–1.03, p=0.02; HR=1.01, 95% CI: 1.00–1.02, p=0.04). Kaplan-Meier analysis revealed reduced recurrence-free survival for patients with >20 Pack-Years (p=0.04), while disease-free survival (DFS) probability was approximately 0.75 at 80 months. No significant associations were found for age, sex, tumor stage, or comorbidities (all p>0.05), and no distant metastases were observed.

**Conclusions:**

TLM appears to be an effective treatment for early-stage laryngeal SCC in this cohort, with smoking identified as a critical modifiable risk factor for recurrence. The findings underscore the importance of smoking cessation interventions to enhance outcomes. Given the retrospective and single-center design, conclusions should be interpreted cautiously. Future multi-center studies with extended follow-up and molecular profiling are recommended.

## Introduction

Laryngeal squamous cell carcinoma (SCC) is one of the most common head and neck malignancies, with a global incidence of approximately 3.5 cases per 100,000 individuals, placing a significant burden on healthcare systems (1). 

This disease is predominantly associated with environmental risk factors such as tobacco smoking and alcohol consumption. In contrast, other factors, including chronic vocal abuse, human papillomavirus (HPV) infection, and exposure to environmental pollutants, also contribute to its etiology (2). 

In its early stages (T1-T2), laryngeal SCC has a favorable prognosis due to advancements in diagnostic and therapeutic approaches, including enhanced screening and minimally invasive surgical techniques such as transoral laser microsurgery (TLM) (3). However, disease recurrence remains a significant challenge in patient management, often leading to diminished quality of life, the need for more aggressive treatments, and reduced overall survival rates (4). Transoral laser microsurgery (TLM) is widely utilized for early-stage laryngeal SCC due to its advantages, including reduced complications, preservation of vocal function, and shorter recovery times (5). Studies have demonstrated that TLM achieves local control rates comparable to radiotherapy or open surgery (85–95%) in T1-T2 patients, with the added benefit of better preservation of voice and swallowing functions(6). However, recurrence rates following TLM vary between 15% and 30% across studies, influenced by factors such as tumor characteristics, patient demographics, and environmental exposures (7).Numerous studies have explored risk factors for laryngeal SCC recurrence. Tobacco smoking is a well-established risk factor, not only for disease onset but also for recurrence. Lin et al. (2025) reported that smoking significantly increases the risk of recurrence in patients treated for laryngeal SCC (4). 

Similarly, Brandstorp-Boesen et al. (2017) found that patients with a smoking history exceeding 20 Pack-Years had a higher recurrence rate following TLM (7). Howren et al. (2022) also confirmed an association between smoking and alcohol consumption and early recurrence (8). Additionally, advanced tumor stage (T3-T4), lymph node involvement, and positive surgical margins have been consistently linked to increased recurrence risk in multiple studies (9,10). Anatomical involvement, such as the anterior commissure or supraglottis, has also been associated with recurrence in some reports; for instance, Jang et al. (2025) found that anterior commissure involvement in T1b patients increased recurrence risk(10), whereas Piazza et al. (2021) reported no such association in T1a patients (6).

Despite these advances, significant gaps in knowledge regarding laryngeal SCC recurrence persist, which and the present study aims to address them. First, most studies on recurrence risk factors have been conducted in Western populations, with limited data from the Middle East, particularly Iran (11). 

Region-specific environmental and cultural factors, such as opium use, which is prevalent in some parts of Iran, may play a distinct role in the recurrence of laryngeal SCC, yet this remains underexplored (12). Second, the impact of less-studied factors, such as chronic vocal abuse, comorbidities (e.g., diabetes, cardiovascular disease), and surgical type (e.g., cordectomy vs. extended laryngectomy), on recurrence remains unclear due to conflicting findings across studies (13,14). Third, the role of molecular biomarkers (e.g., p53, EGFR) in predicting recurrence after TLM remains incompletely elucidated, despite their potential to enhance personalized treatment strategies (15). Finally, there is a paucity of data on the impact of follow-up duration and patterns of early recurrence in TLM-treated patients in Middle Eastern referral centers, underscoring the need for region-specific investigations. This study seeks to investigate the recurrence rate of laryngeal SCC and identify associated risk factors in patients treated with TLM at a referral center in Iran. By focusing on the Iranian population and examining region-specific environmental factors (e.g., smoking, opium use), clinical factors (e.g., tumor grade, anatomical involvement), and surgical factors (e.g., type of TLM), this study aims to address existing knowledge gaps and provide valuable insights for improving treatment management and preventing recurrence. Additionally, it explores patterns of early recurrence and the impact of follow-up duration to inform post-treatment monitoring protocols.

## Materials and Methods

### Study Design

This retrospective cohort study evaluated patients with laryngeal squamous cell carcinoma (SCC) who underwent transoral laser microsurgery (TLM) between July 2016 and July 2022 at Rasoul Akram Hospital, a referral center in Tehran, Iran. The study aimed to assess the disease recurrence rate and identify associated risk factors. Ethical approval was obtained from the Institutional Review Board of Iran University of Medical Sciences (Approval ID: IR.IUMS. REC. 1399. 542). Given the study’s retrospective nature, informed consent was waived.

### Inclusion and Exclusion Criteria

Patients with pathologically confirmed laryngeal SCC in stages T1–T3 who received TLM as their primary treatment were included. Exclusion criteria comprised: 1) patients with distant metastases at initial diagnosis; 2) patients who received concurrent treatments (e.g., radiotherapy or chemotherapy); 3) patients with a prior history of treatment for laryngeal SCC; 4) patients with incomplete medical records; and 5) patients with follow-up less than 12 months, unless recurrence was confirmed earlier.

### Data Collection

Data were extracted from electronic and paper-based medical records at Rasoul Akram Hospital and independently reviewed by two researchers to ensure accuracy and completeness. Collected variables included demographic characteristics (age, sex), environmental risk factors (smoking history in Pack-Years, alcohol consumption, opium use, chronic vocal abuse), clinical characteristics (comorbidities such as diabetes, hypertension, cardiovascular disease), tumor characteristics (tumor grade: T1a, T1b, T2, T3; anatomical involvement: glottis, supraglottis, transglottis, true vocal cord, anterior commissure, false vocal cord), and follow-up data (follow-up duration in months, recurrence status confirmed by biopsy or imaging). Surgical procedures were classified according to the European Laryngological Society (ELS) cordectomy classification (Types I–V). Recurrence was defined as the reappearance of the tumor at the primary site or regional lymph nodes, confirmed by pathological biopsy or imaging (e.g., CT scan or PET-CT).

### Surgical Procedure

Experienced otolaryngologists performed all TLM procedures at the center. A Lumenis UltraPulse CO2 laser with an output power of 10–30 watts and pulsed mode settings was used. This device, operating at a wavelength of 10.6 micrometers and equipped with a microscopic guidance system, ensured high precision in cutting and ablating lesions. Laser intensity and pulse type were adjusted based on the lesion’s depth and location. 

However, complete margin status was not available for all patients due to the dataset’s retrospective nature. Only partial information existed for some cases, which limited the ability to evaluate margin involvement systematically. Surgical types were classified according to ELS cordectomy Types I–V. 

All patients underwent general anesthesia with tracheal intubation to ensure safety and adequate access to the laryngeal region. Surgical margins were assessed in the pathological specimens, though margin status was not available for all patients due to limitations in the retrospective data.

### Statistical Analysis

Statistical analyses were conducted using SPSS version 26 (IBM Corp., Armonk, NY, USA). Continuous variables were reported as mean ± standard deviation (for normally distributed data) or median [interquartile range] (for non-normally distributed data). Categorical variables were presented as counts (percentages). Comparisons between recurrence and non-recurrence groups were performed using the independent t-test or Mann-Whitney U test for continuous variables and the chi-square test or Fisher’s exact test for categorical variables. 

Univariate logistic regression was used to identify risk factors for recurrence, with odds ratios (OR) and 95% confidence intervals (CI) reported. Variables with p<0.1 in univariate analysis were considered for inclusion in a multivariate logistic regression model; however, due to the limited number of recurrence cases, only univariate results were reported. 

Time to recurrence was analyzed using Cox regression. Disease-free survival (DFS) was defined as the time from surgery to recurrence. Overall survival (OS) data were unavailable; thus, analyses refer solely to DFS.

### Ethical Considerations

All data were anonymized to protect patient privacy. The study adhered to the principles of the Declaration of Helsinki, and no interventions were performed on patients as part of this research.

## Results

### Patient Characteristics

This retrospective cohort study enrolled 142 patients with laryngeal squamous cell carcinoma (SCC) treated with transoral laser microsurgery (TLM) at Rasoul Akram Hospital between 2016 and 2022. Recurrence was observed in 26.1% (n=37) of patients, with patterns and timing detailed in the study.

Comparisons between recurrence (n=37) and non-recurrence (n=105) groups revealed significant differences in follow-up duration (p<0.001), with other variables showing varying trends. Full details are provided in Table 1.

**Table 1:** Comparison of Patient Characteristics by Recurrence Status

**Variable**	**Total** ** (n=142)**	**With Recurrence (n=37)**	**Without ** **Recurrence (n=105)**	**p-value**
Age, mean ± SD (years)	61.89 ± 10.79	63.51 ± 9.16	61.32 ± 11.28	0.28
Sex (Male), % (n)	96.5 (138)	100 (37)	95.3 (101)	0.32
Smoking, % (n)	70.6 (101)	73.0 (27)	69.8 (74)	0.87
Pack-Years, median [IQR]	20 [0–40]	35 [0–50]	20 [0–33.75]	0.05
Alcohol Use, % (n)	1.4 (2)	0.0 (0)	1.9 (2)	>0.99
Glottic Involvement, % (n)	97.9 (140)	100 (37)	97.2 (103)	0.56
Supraglottic Involvement, % (n)	14.7 (21)	10.8 (4)	16.0 (17)	0.59
Transglottic Involvement, % (n)	12.6 (18)	10.8 (4)	13.2 (14)	>0.99
True Vocal Cord Involvement, % (n)	97.2 (139)	97.3 (36)	97.2 (103)	>0.99
Anterior Commissure Involvement, % (n)	42.7 (61)	43.2 (16)	42.5 (45)	>0.99
False Vocal Cord Involvement, % (n)	11.2 (16)	10.8 (4)	11.3 (12)	>0.99
Vocal Abuse, % (n)	24.5 (35)	21.6 (8)	25.5 (27)	0.80
Hypertension, % (n)	18.9 (27)	21.6 (8)	17.9 (19)	0.80
Cardiovascular Disease, % (n)	14.0 (20)	21.6 (8)	11.3 (12)	0.20
Diabetes, % (n)	11.9 (17)	8.1 (3)	13.2 (14)	0.56
Opium Use, % (n)	16.8 (24)	16.2 (6)	17.0 (18)	>0.99
Tumor Grade T1a, % (n)	58.0 (83)	54.1 (20)	59.4 (63)	0.70
Tumor Grade T1b, % (n)	23.8 (34)	29.7 (11)	21.7 (23)	0.44
Tumor Grade T2, % (n)	16.1 (23)	16.2 (6)	16.0 (17)	>0.99
Tumor Grade T3, % (n)	1.4 (2)	0.0 (0)	1.9 (2)	>0.99
No Surgical Findings, % (n)	93.7 (134)	97.3 (36)	92.5 (98)	0.44
Paraglottic Involvement, % (n)	4.2 (6)	2.7 (1)	4.7 (5)	>0.99
Cordectomy, % (n)	7.7 (11)	10.8 (4)	6.6 (7)	0.47
Laryngectomy, % (n)	74.8 (107)	75.7 (28)	74.5 (79)	>0.99
Extended Laryngectomy, % (n)	15.4 (22)	13.5 (5)	16.0 (17)	0.91
Follow-up (months), median [IQR]	60 [48–78]	15 [9–27]	66 [54–84]	<0.00
				

### Risk Factors for Recurrence

 Univariate logistic regression identified a significant association with one variable (p=0.02), as summarized in Table 2. 

Cox regression analysis further confirmed this association (p=0.04), with additional results in Table 3.

**Table 2:** Univariate Logistic Regression for Predicting Recurrence Risk

**Variable**	**OR (95% CI)**	**p-value**
Age	1.02 (0.98–1.06)	0.29
Smoking	1.17 (0.51–2.69)	0.72
Pack-Years	1.02 (1.00–1.03)	0.02
Supraglottic Involvement	0.63 (0.20–2.02)	0.44
Transglottic Involvement	0.80 (0.24–2.59)	0.71
True Vocal Cord Involvement	1.05 (0.11–10.40)	0.97
Anterior Commissure Involvement	1.03 (0.49–2.20)	0.93
False Vocal Cord Involvement	0.95 (0.29–3.15)	0.93
Vocal Abuse	0.81 (0.33–1.98)	0.64
Cardiovascular Disease	2.16 (0.81–5.80)	0.13
Diabetes	0.58 (0.16–2.14)	0.41
Laryngectomy vs. Cordectomy	1.06 (0.45–2.54)	0.89
Stage 2 vs. Stage 1	0.95 (0.34–2.60)	0.91
Tumor Grade T1b vs. T1a	1.53 (0.66–3.55)	0.33

**Table 3:** Cox Regression Analysis for Time to Recurrence

**Variable**	**HR (95% CI)**	**p-value**
Age	1.01 (0.99–1.04)	0.42
Smoking	1.09 (0.59–1.99)	0.79
Pack-Years	1.01 (1.00–1.02)	0.04
Supraglottic Involvement	0.74 (0.33–1.68)	0.48
Transglottic Involvement	0.84 (0.36–1.96)	0.69
True Vocal Cord Involvement	0.93 (0.17–5.07)	0.93
Hypertension	1.14 (0.58–2.26)	0.70
Cardiovascular Disease	1.60 (0.78–3.28)	0.19
Stage 2	0.97 (0.46–2.02)	0.93
Extended Laryngectomy	0.36 (0.02–7.82)	0.51
Tumor Grade T1b	1.20 (0.65–2.22)	0.56

### Kaplan-Meier Survival Analysis Based on Smoking Exposure

**Fig 1:**
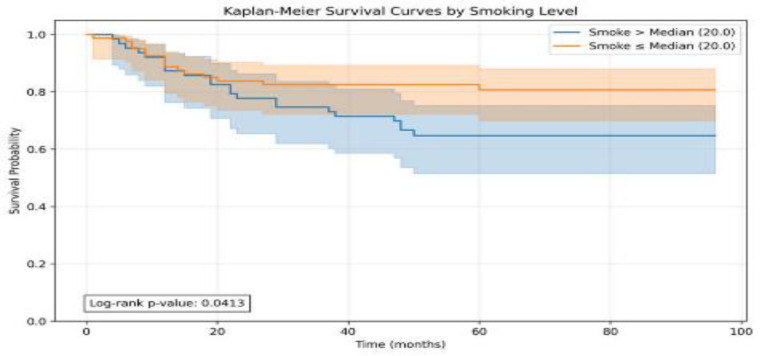
Kaplan–Meier survival curve based on the level of cigarette consumption in patients with laryngeal SCC undergoing TLM surgery.

 The Kaplan-Meier survival curve, stratified by smoking exposure using a median cutoff of 20 Pack-Years, is depicted in Figure 1. Patients with smoking above the median (blue line) exhibited significantly lower recurrence-free survival compared to those at or below the median (orange line). The log-rank test yielded a p-value of 0.04 (p<0.05), indicating a statistically significant association between higher smoking exposure and reduced recurrence-free survival in SCC patients post-TLM.

**Fig 2:**
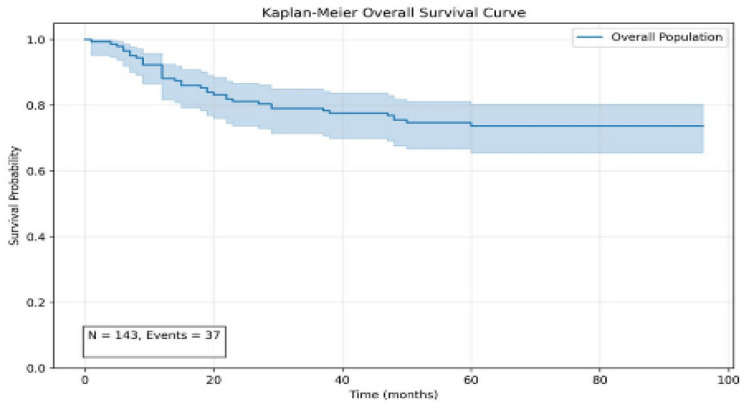
Kaplan–Meier curve for overall survival in the entire patient population (n = 142).

### Overall Survival Analysis Using the Kaplan-Meier Method

 The overall survival curve for the entire cohort (n=142) is presented in Figure 2, based on Kaplan-Meier analysis. A total of 37 well-defined events were recorded, consisting of 17 deaths and 20 recurrence-related events. The x-axis represents follow-up time in months, and the y-axis indicates the cumulative survival probability. The curve shows a gradual decline, with survival probability remaining approximately 0.75 at 80 months. The 95% confidence interval, shaded in blue, underscores the statistical precision of the survival estimates.

## Discussion

This study investigated 142 patients with laryngeal squamous cell carcinoma (SCC) treated with transoral laser microsurgery (TLM) at Rasoul Akram Hospital (2016–2022), revealing a 26.1% recurrence rate, a significant association between smoking (Pack-Years) and recurrence (OR=1.02, p=0.02; HR=1.01, p=0.04), and Kaplan-Meier analyses indicating reduced recurrence-free survival with smoking >20 Pack-Years (p=0.0413) and a 0.75 overall survival probability at 80 months.

The observed recurrence rate of 26.1% falls within the range reported in prominent Q1 studies. Brandstorp-Boesen et al. (2016) documented a 23% recurrence rate in a large Norwegian cohort of 1,616 patients over 27 years, with the majority occurring within the first 3 years, closely aligning with our median time to recurrence of 15 months(16). Similarly, Valls-Mateus et al. (2016) (17) reported a 24% recurrence rate in 163 TLM-treated patients, with a predominance of local recurrences, mirroring our 70.3% local recurrence rate, likely attributable to the early-stage predominance (81.8% T1-T2) in both studies, which limits the scope for regional or distant spread. However, the absence of distant metastases in our cohort contrasts with Zhang et al. (2021), who identified a 2.12% lung metastasis rate in a SEER-based analysis of 10,935 patients(15), and Shin et al. (2023)(18), who reported a 5% distant metastasis rate in 1,562 European cases. This discrepancy may be attributed to our shorter median follow-up of 60 months compared to the more extended observation periods in these studies (up to 2014 for Zhang et al. and multi-year follow-ups for Shin et al.), as well as our exclusion of advanced-stage patients (T4 or N+), who are more prone to metastatic dissemination.

The significant association between smoking exposure and recurrence risk is a cornerstone finding, corroborated by multiple high-impact studies. Kim et al. (2023)(19) conducted a meta-analysis of 32,128 patients across 45 studies, establishing smoking as a dose-dependent risk factor with hazard ratios that escalate with cumulative exposure, a pattern consistent with our Pack-Year results (p=0.04). The Kaplan-Meier analysis (p=0.0413) further supports this, aligning with Valls-Mateus et al. (2016)(17), who reported a hazard ratio of 1.8 for smokers in a 417-patient Italian cohort. However, their 10-year smoking threshold differs from our 20-pack-year median, reflecting variations in exposure prevalence and cohort demographics. In contrast, Zhang et al. (2021) (15) highlighted alcohol consumption as a stronger predictor in a Chinese cohort, and Abou-Foul et al. (2024) (20)found smoking less influential in advanced stages, suggesting that regional lifestyle factors (e.g., higher alcohol use ) or stage-specific treatment responses may modulate these associations. Our finding of a higher median Pack-Years in the recurrence group (35 vs. 20, p=0.058) supports a dose-response relationship, warranting further exploration of smoking cessation interventions.

The lack of significant differences in age, sex, tumor stage, and comorbidities between recurrence and non-recurrence groups presents an interesting divergence from the literature. Fong et al. (2019)(21) identified age ≥60 years and TNM stages III-IV as predictors of poorer disease-free survival in a multi-center study of 1,200 patients, a contrast potentially explained by our cohort’s younger mean age (61.89 years) and early-stage focus (T1-T2: 81.8%), which may dilute age-related effects. Leoncini et al. (2018)(22) emphasized nodal status as a critical prognosticator in a 33,000-patient INHANCE consortium study, which differs from our all-N0 cohort, reflecting our exclusion of node-positive cases. The non-significant role of opium use (p>0.999) contrasts with Talamini et al. (2002)(23), who reported a synergistic effect with tobacco in 527 Italian cases, and Singh et al. (2021)(24), who linked opium to increased head and neck cancer risk in a 50,000-patient study. This discrepancy may arise from our smaller sample size, the lack of detailed quantification of opium exposure, or regional variation in usage patterns, necessitating standardized assessment in future research.

The Kaplan-Meier survival analyses provide additional prognostic insights. The recurrence-free survival difference by smoking status (p=0.0413) is consistent with Tirelli et al. (2024)(25), who reported a similar trend, though their inclusion of advanced stages may explain slight differences. The overall survival probability of 0.75 at 80 months aligns with Breda et al. (2025)(26), reported a 65% five-year survival in 520 patients, and Vilaseca et al. (2015)(27), who recorded 70% at five years in TLM cases. However, our uniform survival trend across stages differs from their observations of worse outcomes in T3-T4 cases, likely because we used TLM exclusively, whereas they used mixed modalities (e.g., radiotherapy/chemoradiotherapy), highlighting TLM’s efficacy in early-stage disease. This is further supported by Succo et al. (2019)(28), who validated TLM’s organ-preservation benefits in a 1,000-patient European cohort.

Several strengths enhance the robustness of this study, including the detailed clinical data collection and the use of TLM, a technique endorsed by Succo et al. (2019)(28) for its oncologic and functional outcomes. Nevertheless, limitations must be acknowledged. The single-center design and median follow-up of 60 months may constrain generalizability and the detection of late recurrences or metastases, as evidenced by the longer follow-up in Zhang et al. (2021)(15). The absence of human papillomavirus (HPV) status and molecular markers (e.g., p53 mutations, Nylander et al., 1997(29)) limits the ability to assess their prognostic roles, a gap noted in studies. The cohort’s early-stage predominance may not fully capture advanced disease dynamics, and the lack of standardized opium use data may underestimate its impact.

Importantly, surgical margin status was not available for all patients, which represents a significant limitation. Margin involvement is a well-established prognostic factor in laryngeal cancer, and incomplete margin data restricted our ability to incorporate this parameter into risk modeling or to adjust for it in survival analysis. Furthermore, because complete data on several key confounders—including margin status—were missing, multivariate regression could not be reliably performed. As a result, the associations identified in univariate analyses may be influenced by residual confounding (e.g., age, tumor stage, or comorbidities), and findings should therefore be interpreted with caution. Future studies with comprehensive data collection are needed to enable fully adjusted multivariable models. Future studies should adopt multi-center designs, incorporate molecular profiling (e.g., HPV, p53), extend follow-up beyond 5 years, and employ validated tools to quantify regional risk factors, such as opium use. Additionally, integrating smoking cessation programs and personalized treatment algorithms based on exposure levels could optimize outcomes, addressing the dose-response relationship observed herein.

## Conclusion

This study suggests that transoral laser microsurgery (TLM) may be a practical approach for managing early-stage laryngeal squamous cell carcinoma in this cohort. However, the retrospective and single-center design limits the strength of this conclusion. The results indicate that smoking exposure is a potentially important factor associated with recurrence risk, rather than definitively establishing causality. These findings may support the potential value of smoking cessation counseling and tailored follow-up strategies, but further multi-center prospective studies are required to confirm these observations. Overall, the study provides preliminary evidence to guide future research toward more individualized care protocols.
